# Cell-type specific metabolic profiling of *Arabidopsis thaliana* protoplasts as a tool for plant systems biology

**DOI:** 10.1007/s11306-015-0814-7

**Published:** 2015-06-06

**Authors:** Sara V. Petersson, Pernilla Lindén, Thomas Moritz, Karin Ljung

**Affiliations:** 1 Umeå Plant Science Centre, Department of Forest Genetics and Plant Physiology, Swedish University of Agricultural Sciences, 901 83 Umeå, Sweden; 2 BioVis, Department for Immunology, Genetics, and Pathology, Uppsala University Rudbecklaboratoriet, 751 85 Uppsala, Sweden

**Keywords:** Metabolite profiling, Untargeted metabolomics, Gas chromatography-mass spectrometry, *Arabidopsis thaliana*, Flow cytometry, Multivariate statistical analysis

## Abstract

Flow cytometry combined with cell sorting of protoplasts has previously been used successfully for transcript profiling of the *Arabidopsis thaliana* root. We have developed the technique further, and in this paper we present a robust and reliable method for metabolite profiling in specific cell types isolated from *Arabidopsis* roots. The method uses a combination of fluorescence-activated cell sorting and gas chromatography-time of flight-mass spectrometry analysis. Cortical and endodermal cells from the green fluorescent protein (GFP)-expressing enhancer trap line J0571 were analysed and compared with non-GFP-expressing cells and intact root tissue. Of the metabolites identified, several showed significant differences in concentration between cell types. Multivariate statistical analysis was used to compare metabolite patterns between cell and tissue types, showing that the patterns differed substantially. Isolation of specific cell populations combined with highly sensitive MS-analysis will be a powerful tool for future studies of plant metabolism, and can also be combined with transcript and protein profiling for in-depth analyses of cellular processes.

**Electronic supplementary material:**

The online version of this article (doi:10.1007/s11306-015-0814-7) contains supplementary material, which is available to authorized users.

## Introduction

Plants are highly complex multicellular organisms comprised of multiple specialised and integrated organs and tissues. Different cell types have distinct transcript, protein and metabolite contents, enabling them to carry out specific activities and ensure that the function of each tissue is integrated with that of the plant as a whole. Despite the complexity of plant structure, most “omics” analyses are performed on whole plants or whole plant organs, undoubtedly losing levels of detail in the process.

The development of new types of mass spectrometers with increasingly greater sensitivity and resolution means that it is now possible to quantify metabolites in much smaller amounts of plant material than in the past, while maintaining both accuracy and precision in the analyses (Lee et al. 2012; Obata and Fernie 2012). It is now feasible to acquire detailed information about metabolite composition from small amounts of plant tissue, and even from isolated cells (Rubakhin et al. 2013; Misra et al. 2014). In order to take full advantage of these sensitive analyses, the sampling techniques used also need to be improved. Cell type-specific sampling techniques have usually involved laborious dissection of plant tissues; using techniques like cryo-sectioning or laser capture micro-dissection (LCM) (reviewed in Wang et al. 2012). Schrader et al. (2004) used cryo-sectioning of the vascular cambium of *Populus tremula* stems to create a high-resolution transcriptional map across the wood-forming meristem. LCM has been employed in combination with GC-TOF–MS for metabolite profiling of *Arabidopsis thaliana* vascular bundles (Schad et al. 2005). LCM combined with mass spectrometry has also been used to analyse the distribution of tissue-specific secondary metabolites in rapeseed (*Brassica napus)* (Fang et al. 2012). Cryo-sectioning and LCM have proved useful for tissue and cell-specific analysis, but they are time-consuming and of limited use for collection of very small and complex three-dimensional structures.

Flow cytometry combined with cell sorting [also called fluorescence-activated cell sorting (FACS)] has recently emerged as a powerful and versatile technique for isolating specific cell types from complex plant tissues (reviewed in Wang et al. 2012 and Carter et al. 2013). FACS has mainly been used in mammalian systems where separating cells is relatively straightforward. Plant tissues are more difficult to work with, requiring enzymatic degradation of the cell wall to isolate free protoplasts from the tissues under investigation (Dolezel et al. 2007). Collections of transgenic *Arabidopsis* lines that express green fluorescent protein (GFP) in specific cell types (Haseloff 1999) have been used successfully for protoplasting and cell sorting of plant tissues by FACS (Birnbaum et al. 2005). Using these transgenic *Arabidopsis* lines, it has been possible to isolate specific cell types from within complex three-dimensional structures such as root (Birnbaum et al. 2003) and shoot (Yadav et al. 2009) apices. To date, the technique has mainly been applied to transcript profiling of specific root cell types (Birnbaum et al. 2003; Brady et al. 2007), but it has also been used for analysis of cell type-specific proteins (Petricka et al. 2012) and metabolites (Moussaieff et al. 2013; Pěnčík et al. 2013; Petersson et al. 2009). Moussaieff et al. (2013) describe a method for metabolite profiling of sorted protoplasts using LC–MS/MS, while targeted approaches for analyses of growth regulatory compounds using LC- and GC–MS/MS was described in Pěnčík et al. (2013) and Petersson et al. (2009).

Here we describe further development of the technique for cell type-specific metabolite profiling, based on FACS in combination with GC-TOF–MS analysis. The method was used for metabolite profiling of the transgenic *Arabidopsis* line J0571, which expresses GFP in the cortical and endodermal cell files of the root tip. Using multivariate statistical analysis (MVA), we were able to separate the metabolite profiles of the different cell types, and we could also show that the concentrations of specific metabolites differed between these cell types. The method for protoplast isolation and analysis is very robust, sensitive and accurate, and it can be used in combination with different mass spectrometry techniques to profile the metabolite content in specific root cell types. It can also be combined with transcript and protein analyses, as a tool for plant systems biology at the cellular level.

## Materials and methods

### Plant material and growth conditions

*Arabidopsis thaliana* seeds of the wild-type line C24 and the GFP-expressing line J0571 (Haseloff 1999) were surface sterilized, sown on plates containing solid media (1× Murashige Skoog, 1 % sucrose (Duchefa), 1 % agar (Merck, NJ, USA), pH 5.8) covered with a nylon mesh (Sefar Nitex 03-110/47), vernalized for 2 days at 4 °C and then grown vertically for 10 days under long day conditions (8 h darkness/16 h light, 150 µE, 22 °C). For each sorting experiment, 20 plates were sown with C24 or J0571 seeds (three rows with 70 µl seeds/plate).

### Chemicals

If not otherwise stated, the chemicals used were purchased from Sigma-Aldrich (St Louis, MO, USA) at the highest available purity.

### Laser confocal microscopy

Confocal images of *Arabidopsis* roots were captured using a Zeiss 710 NLO two-photon confocal microscope equipped with a 488 nm laser, and the pictures were analysed using the Zeiss ZEN software package (Zeiss, Oberkochen, Germany).

### Protoplast isolation

Protoplast isolation was done according to Petersson et al. (2009), with minor modifications. The apical third of the roots from 10-day-old seedlings were collected and incubated in an enzyme solution containing pectolyase (Sigma-Aldrich P-5936) and cellulysin (Calbiochem 219466) dissolved in buffer (600 mM mannitol, 2 mM MgCl_2_, 0.1 % BSA, 2 mM CaCl_2_, 2 mM MES and 10 mM KCl, pH 5.7) to a final concentration of 45 units/ml cellulysin and 0.3 units/ml pectolyase. The enzyme treatment was done for 1.5 h in the dark at room temperature with gentle shaking at 130 rpm.

The protoplast suspension was filtered through a 40 µm cell strainer (BD Falcon) to remove root tissue and debris, and immediately centrifuged at 1000 rpm for 3 min. The supernatant was discarded, and the protoplast pellet was suspended in 1.5 ml 1× PBS (0.15 M NaCl, 8 mM Na_2_HPO_4_, 2.7 mM KCl, and 1.47 mM KH_2_PO_4_, pH 7.4). The protoplasts were kept at 4 °C during the sorting procedure.

### Flow cytometry and cell sorting

Protoplasts were analysed and sorted by FACS on a BD FACSAria I, equipped with 405, 488 and 633 nm lasers, using the FACSDiva 6.0 software (BD Biosciences, Erembodegem, Belgium). The BD FACS Flow sheath fluid was replaced with 0.7 % NaCl solution in order to reduce interference from phosphates and other compounds during the MS analysis. GFP+ and GFP− protoplast populations were identified based on their forward and side scatter, their GFP intensities detected using the LP502 and BP(539/30) filter after excitation with the 488 nm laser, and their autofluorescent properties detected using the LP600 and BP(610/20) filter after excitation with the 561 nm laser. Nozzle aperture was set to 100 µm, sheath gas pressure 20 psi, sorting rate 2000–3000 events/s. The protoplasts were sorted into 15 ml Falcon tubes, and each sample contained approximately 1 million protoplasts. The yield from sorting the J0571 line was normally 1 million GFP+ and 4–5 million GFP− protoplasts, and that from sorting the C24 line was 5–6 million GFP− protoplasts.

The sorted cells were centrifuged at 1500 rpm (Hettich Universal 32, Germany) for 15 min. The supernatant was discarded and the cells were frozen immediately in liquid nitrogen and stored at −80 °C until required for extraction.

### Metabolite profiling

The frozen protoplast sample, containing around 1 million protoplasts in approximately 100 µl 0.7 % NaCl solution, was thawed on ice. Samples were extracted as described by Gullberg et al. (2004), with modifications to take into account the small amount of tissue. One ml extraction mixture (MeOH:CHCl_3_:H_2_O, 1:1:3) containing 120 ng/µl of each internal standard (l-proline-^13^C_5_, succinic acid-D_4_, salicylic acid-D_6_, alpha-ketoglutarate-^13^C_4_, glutamic acid-^13^C_5_, putrescine-D_4_, myristic acid-^13^C_3_, d-glucose-^13^C_6_, hexadecanoic acid-^13^C_4_, sucrose^13^C_12_, cholesterol-D_7_) was added. The sample was sonicated for 5 min and centrifuged at 2500 rpm for 5 min, and the supernatant was transferred to a 5 ml glass vial and evaporated to dryness without heating (approximately 30 °C). The sample was then resuspended in 150 µl analytical grade MeOH, transferred to a GC vial and evaporated to dryness. This was repeated with an additional 150 µl MeOH, which was added to the GC vial and then again evaporated to dryness. Eight µl of methoxyamine in pyridine (15 ng/µl) was added to the dry sample, and the sample was incubated for 16 h at 25 °C. Eight µl MSTFA (Thermo Scientific, Waltham, MA, USA) was then added, and the sample was incubated for 1 h at 25 °C. Finally, 8 µl of methyl stearate in heptane (15 ng/µl) was added prior to GC-TOF–MS analysis.

In addition to experimental samples, quality control samples, blank samples and an n-alkane series (C8–C40), used to determine retention indices, were included in the analysis (Schauer et al. 2005). One μl of derivatized sample was injected into a split/splitless injector in splitless mode, by an CTC PAL systems auto sampler with a 10 μl syringe, in an Agilent technologies 7890A GC system (Agilent Technologies, Atlanta, GA, USA) equipped with a 30 m × 0.250 mm diameter fused silica capillary column with a bonded 0.25 μm Durabond DB-5 ms UI stationary phase (part no: 122-5222UI, Agilent J&W GC columns). The injector temperature was 260 °C, and the front inlet septum purge flow was 3 ml/min. The gas flow rate through the column was 1 ml/min; column temperature was held at 70 °C for 2 min, then increased by 20 °C/min to 320 °C, and held for 8 min. The column effluent was introduced into the ion source of a Pegasus HT GC, high throughput TOF–MS (LECO Corp., St Joseph, MI, USA) operating in electron impact ionization (EI) mode. The transfer line and ion source temperatures were 270 and 200 °C respectively. Detector voltage was 1500 V and ions were generated by a −70 V electron beam at an ionization current of 2.0 mA; 20 spectra/s were recorded in the mass range 50–800 m/z. The acceleration voltage was turned on after a solvent delay of 270 s. All non-processed MS files from the analysis were exported from the ChromaTOF software as NetCDF files to MATLAB (ver: 8.1, MathWorks, Natick, MA, USA) for an untargeted metabolite analysis in which the necessary data pre-treatment procedures such as base-line correction, peak alignment and hierarchical multivariate curve resolution (H-MCR) were performed using custom scripts, essentially according to Jonsson et al. (2005). A targeted metabolite analysis was also performed, using an additional in-house MATLAB script. For this analysis, only metabolites that could be classified and/or identified using a reference spectrum were analysed (Supporting Information Table 1). The spectral and identification information from the in house script used for metabolite identification can be found in Supporting Information Data 1. The result from each of these analyses was in the form of a data table, where each row represented one sample and the columns correspond to the resolved peak area intensities. The sorted cell samples were normalized to the number of cells/sample and the root samples were normalized to mg of tissue/sample. All samples were scaled to the summed intensity of all metabolites in the sample.

### Statistical analysis

All multivariate statistics was performed using the SIMCA software package version 13.0 (Umetrics, Umeå, Sweden) as described by Bylesjö et al. (2006). Before analysis, the samples were scaled to unit variance (UV). A PCA model was built from a number of principal components (PC), where the scores (t) describe the relations between samples and the loadings (p) describe the relations between variables/factors. PCA is an unsupervised method. In PCA, the PCs describe the general properties of data (X) in consecutive order. The variation in data was explained by the model goodness of fit (R^2^X), with values ranging from zero to one. The predictive ability of each model was described by the goodness of prediction (Q^2^), ranging from minus one to one. In general, positive Q^2^ values indicated good predictive ability. The supervised OPLS-DA technique was used to assess the maximum class separations (Bylesjö et al. 2006). The OPLS-DA model was built from both predictive and orthogonal components. For more information regarding all PCA and OPLS-DA models presented, see Supporting Information Data 2.

## Results and discussion

### A rapid and simple method for protoplast isolation and cell sorting

Protoplasting combined with cell sorting has been used primarily as a method to isolate specific cell types for transcript profiling, although there are also examples where the technique has been combined with metabolite analysis (Moussaieff et al. 2013; Pěnčík et al. 2013; Petersson et al. 2009). Moussaieff et al. (2013) pointed out that profiling of metabolites from sorted cells was problematic because of the high concentration of phosphates and other compounds in the sorting buffer. Instead of altering the downstream sample purification process to overcome this problem, and thereby risk losing low-abundance metabolites, we replaced the FACS sheath buffer with a 0.7 % NaCl solution. The NaCl solution worked equally well as a sorting buffer, and the protoplasts remained intact and alive.

As root protoplasts are fragile, and the sorting process can place stress on the cells, a larger nozzle aperture, 100 µm, was preferred to the standard 70 µm aperture. Also, root protoplasts range in size between 5 and 50 µm (Petersson et al. 2009) and a nozzle aperture of at least twice the cell diameter should normally be used. A larger nozzle aperture is always combined with a lower sheath pressure (20 psi) and slower sorting rate (2000–3000 events/s). Although this meant that the protoplasts were surrounded by a greater amount of liquid, serving as a protective shield, it had the consequence that they were suspended in a larger volume of 0.7 % NaCl. Freeze-drying and other methods were initially tested as means to reduce the volume of the sorting buffer and isolate the metabolites, but it was concluded that the easiest and fastest way to reduce the volume was to spin down the protoplasts after sorting and discard the 0.7 % NaCl solution. This eliminated most of the sorting buffer, which enabled us to extract the metabolites with minimal losses, resulting in very high sensitivity and excellent chromatographic separation in the GC-TOF–MS analysis.

**Fig. 1:**
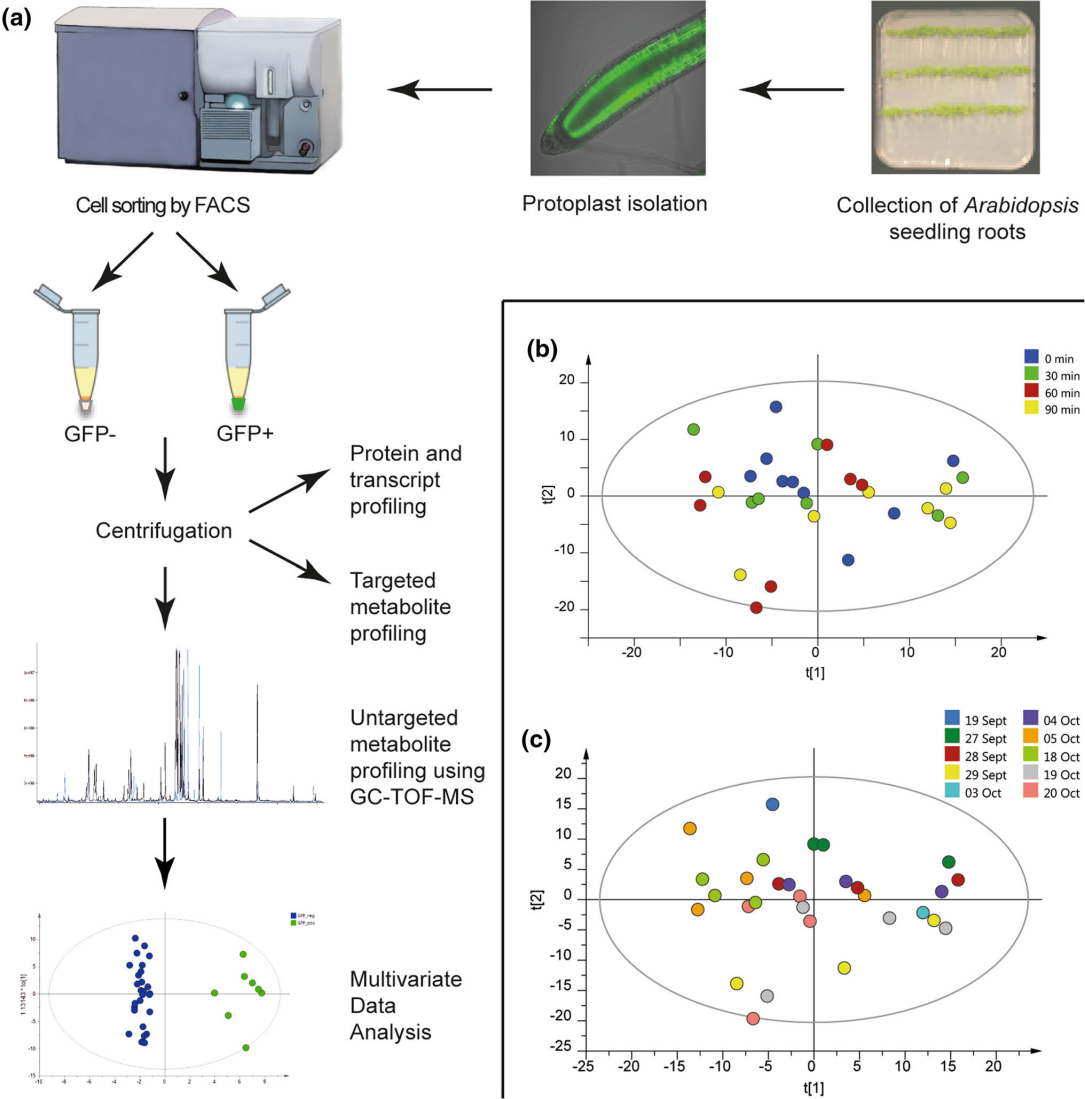
**a** Method overview. *Arabidopsis thaliana* seedlings were grown on vertical agar plates, and root tips were harvested 10 days after germination. Protoplasts were isolated after enzymatic degradation of the cell walls and sorted into GFP− and GFP+ cell fractions using FACS. Intact protoplasts were then collected by centrifugation, and untargeted metabolite profiling was performed using GC-TOF–MS analysis. MVA was used to evaluate the data sets. The collected protoplasts could also be used for targeted metabolite profiling of specific compound classes, as well as transcript and protein profiling. **b** PCA score plot of wild-type data from the control experiment, showing the samples for different technical replicates, coloured according to time after protoplast isolation (sorting started at different times after protoplast isolation). Start of sorting after isolation 0 min (*green*), 30 min (*blue*), 60 min (*red*), 90 min (*yellow*). **c** PCA score plot of wild-type data from the control experiment, showing the samples for different biological replicates, coloured according to collection day. Sorting was performed on 10 different days from September 9th to October 20th. The PCA model for the control experiment was based on six components. n = 30, number of metabolites = 505, R^2^X = 0.735, Q^2^ = 0.557

Taken together, these improvements made the process simpler, faster and gentler than previously published methods, and the method also has the great advantage that the sorted cells can be used not only for metabolite profiling, but also for protein and transcript analyses. See Fig. 1a for an overview of the method.

### Validation of the method for metabolite profiling of sorted plant cells

Protoplasts from *Arabidopsis* wild-type C24 roots were used for development and validation of the technique. Key to the usefulness of the method is the ability to monitor biological variation, as well as changes in metabolite profiles that might occur during the protoplast isolation and sorting process. The enzymatic treatment to isolate free protoplasts takes 1.5 h, and depending on the cell type-specific GFP line used, it normally takes 1 h to sort and collect 1 million GFP+ protoplasts using FACS.

In order to examine whether the duration of sorting by FACS had a significant influence on the metabolite profile, protoplasts isolated from C24 roots were sorted for 30 min as four consecutive samples, with a total sorting time of 120 min (sorting was conducted over 30 min starting at 0, 30, 60 and 90 min after protoplast isolation).

To verify the robustness of the method, C24 protoplasts were also sorted on 10 different days over a period of 3 months. The protoplasts were sorted at the same time each day, keeping the sampling conditions identical in order to minimise any variation in metabolite levels.

The collected fractions, each containing 1 million wild type Arabidopsis protoplasts, were centrifuged and the protoplast pellet was used for GC-TOF–MS metabolite analysis. MVA was then performed on the MS data. Firstly, a principal component analysis (PCA) was done in order to evaluate whether there were any metabolic changes occurring during protoplast sorting (Fig. 1b) or depending on day of sampling (Fig. 1c), based on all detected peaks in the MS-data (putitative derivatised metabolites) (PCA model: components = 6, n = 30, number of peaks = 505, R^2^X = 0.591, Q^2^ = 0.149). No trend related to the time of sorting or the day of sampling could be observed. As a complementary approach, supervised orthogonal partial least square discriminant analysis (OPLS-DA) modelling (Bylesjö et al. 2006) was performed, and no significant models related to the time of sorting or the date of sampling could be fitted. Hence, the method does not appear to suffer from systematic variations.

Based on these experiments and previous controls, described in Petersson et al. (2009), we concluded that the technique was robust and reproducible, and could therefore be used for cell type-specific metabolite analysis of *Arabidopsis* lines.

### Protoplast isolation and sorting of the GFP-expressing line J0571

**Fig. 2:**
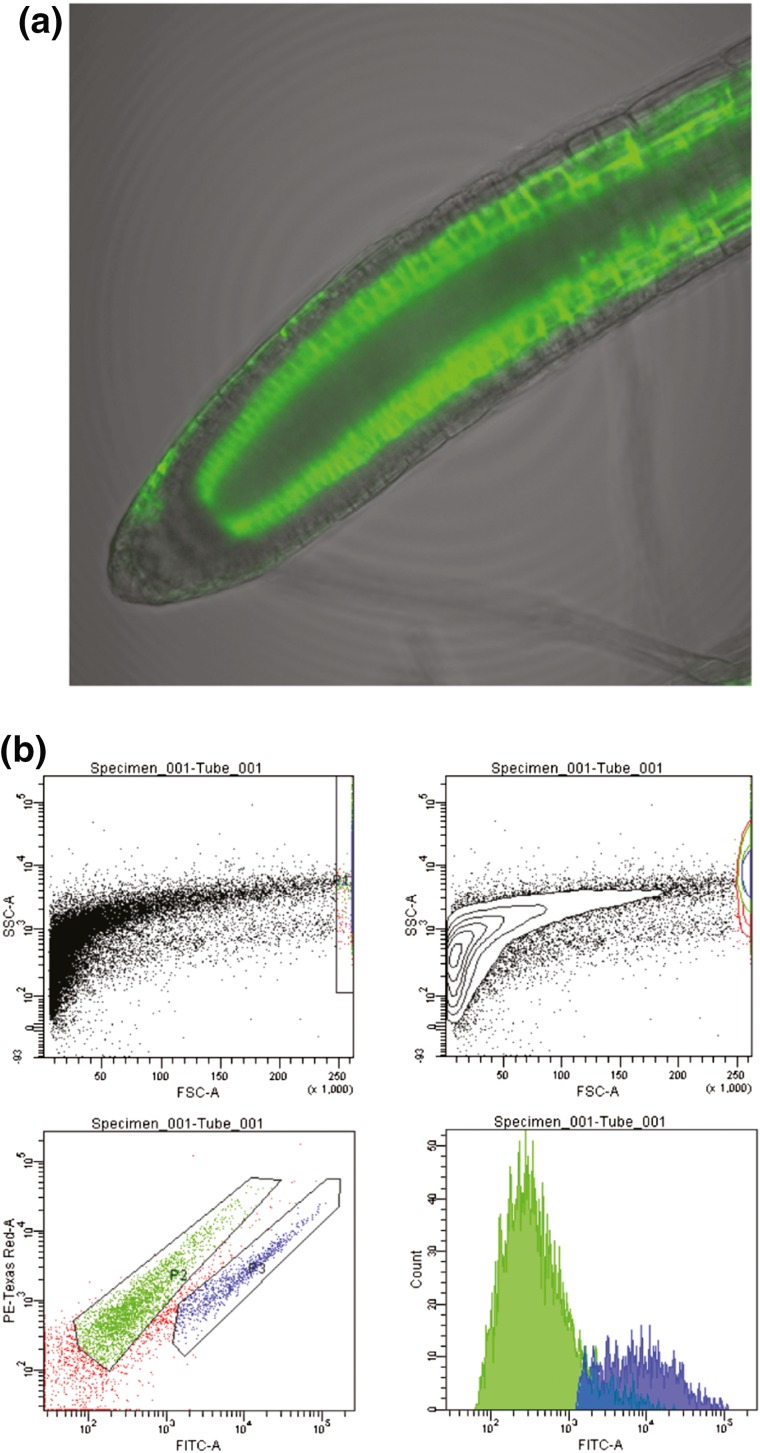
**a** Confocal image of a root tip from the *Arabidopsis* line J0571, showing strong GFP expression in cortical and endodermal cell files. **b** FACS gating of J0571 protoplasts. The pictures show a typical cell sorting experiment (n = 10). Gating was based on forward and side scatter properties and intensity in the FITC (excitation 488 nm and emission filter BP539/30 nm) and Texas-Red® (excitation 488 nm and emission filter BP 610/20 nm) channels in order to analyse and sort the GFP + (blue) and GFP− (*green*) protoplasts. *Top left image* shows all particles, where only the protoplasts and particles of similar size and granularity are gated. As the protoplasts approach the upper size limit for FACS they are distributed along the right FSC axis, which is a relative measure of size.* Top right image* is a density plot of the same image, showing the amount of protoplasts actually present in the sample. The *bottom left image* shows how the final gates were set, according to their GFP intensities and auto-fluorescence in the *red* region. The* bottom right image* shows the intensity distribution of the GFP positive and negative samples, coloured according to gated population

Root tips were excised from 10-day-old *Arabidopsis* seedlings from the enhancer trap line J0571, which expresses GFP exclusively in cortical and endodermal cells in the root apex (Haseloff 1999). The line J0571 has been used in several studies of root development in *Arabidopsis* (Petersson et al. 2009; Sozzani et al. 2010; Ubeda-Tomás et al. 2008), and has a stable and distinct pattern of GFP expression (Fig. 2a). The forward and side scatter, red auto-fluorescence and GFP properties of the protoplasts were analysed by flow cytometry, using a BD FACSAria I cell sorter (Fig. 2b). Protoplasts from root tips of J0571 seedlings grown on 20 agar plates were all sorted within 2 h of isolation. This amount of root tissue gave a yield of around 1 million GFP+ protoplasts and approximately 4–5 million GFP− protoplasts. Each isolation and sorting process took 1 day and were repeated ten times over a 2 months period. After FACS sorting, the protoplasts were centrifuged and the protoplast pellet was frozen in liquid nitrogen and stored at −80 °C. The results of the J0571 experiments were stable from day to day in terms of the isolation efficiency and the percentages of GFP positive and negative protoplasts isolated, and no major adjustments to the gating were required between sorting days.

### Metabolite profiles of root tissue and sorted protoplasts show distinct patterns

**Fig. 3:**
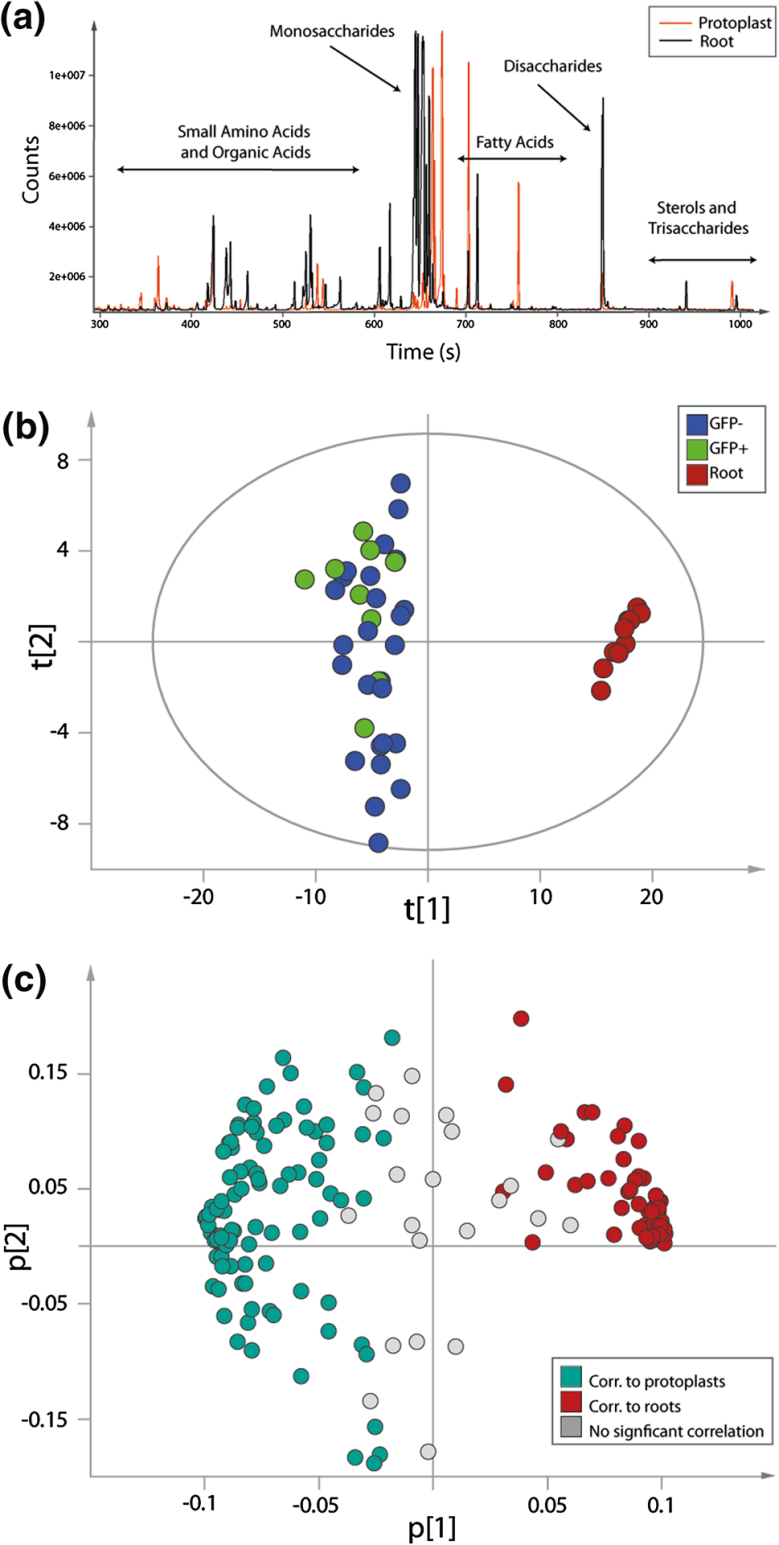
Metabolite profiling of root tissue and sorted protoplasts. **a** Total ion chromatogram (TIC) for root tissue (*black*) and sorted protoplasts (*orange*). The chromatograms show that the two types of sample have different metabolite profiles. **b** The first two components of the PCA score plot from root tissue (*red*) and sorted protoplasts (GFP− protoplasts (*blue*), GFP + protoplasts (*green*)) from the *Arabidopsis* line J0571. **c** Loading plot for the PCA model. Metabolites to the right side of the graph are correlated with root tissue, and metabolites to the left side are correlated with sorted protoplasts from the *Arabidopsis* line J0571

The most apical third of roots from 10-day-old J0571 seedlings were collected, and protoplasts were isolated from the tissue as described in 3.3. Metabolites from isolated and sorted protoplasts (GFP-expressing cortical and endodermal cells and non-GFP-expressing reference cells from the line J0571), as well as from intact root tissues (instantly frozen after sampling and not thawed before metabolite extraction), were analysed using GC-TOF–MS. A comparison of the total ion chromatograms (TIC) showed that metabolites from the protoplasts and from the root tissue samples were in the same concentration range. This made it possible to investigate differences in individual classes of compound between the samples. Differences were found in several compound classes, most likely due to the absence of cell walls in the protoplast samples (Fig. 3a). In order to be able to compare the metabolite content of protoplasts with that of intact roots, the raw data were scaled against the total peak area of each sample. In addition, the root samples were normalised to mg root tissue and the protoplasts to number of cells. In the subsequent PCA, a clear difference between the root tissues and the GFP positive and GFP negative protoplasts was observed (Fig. 3b) (PCA model: components = 5, n = 45, number of metabolites = 175, R^2^X = 0.735, Q^2^ = 0.557). The model was based on identified metabolites only.

From the corresponding loadings, we concluded that plant sterols and long-chain carbon compounds were prominent in protoplasts while there were more sugars and amino acids in the roots (Table 1). The majority of the metabolites in the loading plot clearly grouped with either roots (red) or protoplasts (turquoise) (Fig. 3c). For a complete list of metabolites see Supporting Information Table 1.

**Table 1:** PCA model over roots and sorted protoplasts. Of the 45 samples analysed, 10 were from intact root and 35 were protoplast samples

The model was based on 175 metabolites. Nine unknowns were not included in the table since they had no significant correlation with either tissue type (see Supporting Information Table 1). Thirty eight metabolites were correlated with roots and 66 with protoplasts (confidence level of parameters 99%). The grey scale to the right of the metabolites indicates level of correlation. Dark grey represents the highest correlation and light grey the lowest (but still significant) correlation. See Fig. 3b for the score plot and Fig. 3c for the loading plot for the PCA model

The most obvious difference in metabolite profile between the intact root samples and the sorted protoplasts was the change in carbohydrate profile. The protoplast samples contained many unidentified long-chain carbon compounds that were not observed in the root samples. We were unable to positively identify them, but it is likely that they originated from the plasma membrane. It is possible that these metabolites were not extracted by the current method used for extraction of intact roots, or that they constitute only a minor metabolite fraction, and were therefore not detected in the whole root samples.

### The metabolome of the cortex and endodermis cell types is distinct from that of the reference cells

**Fig. 4:**
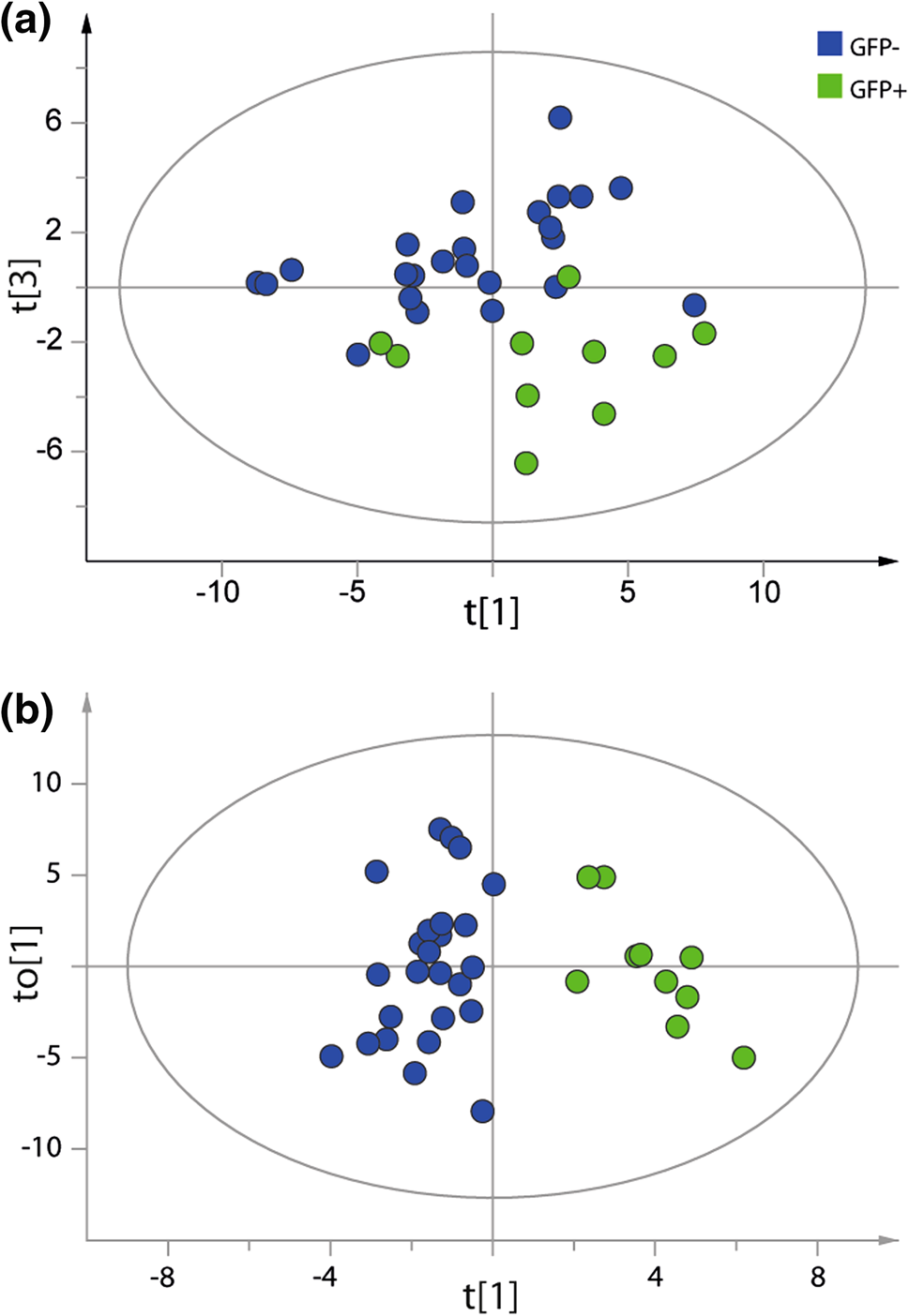
Metabolite profiling of sorted GFP+ (*green*) and GFP− (*blue*) protoplasts based on the metabolic profiles from the identified metabolites. **a** PCA model of sorted protoplasts. Separation between GFP + and GFP− protoplasts was observed according to the t[1]/t[3]-score plot. **b** OPLS-DA modeling of sorted GFP + against GFP− protoplasts. The model was based on the metabolites identified. 38 % of the variation in X (R^2^X) could be related to 85 % of the classification described by the Y-variable. The model also had good predictive ability (Q^2^)

Root samples were excluded from the data set in order to investigate possible differences between the GFP+ protoplasts and the GFP− reference cells isolated from J0571 seedling roots. The data set was reduced to only those metabolites that could be classified (n = 45, variables = 58). A separation between GFP+ and GFP− protoplasts was observed according to the t[1]/t[3]-scores plot based on the metabolic profiles of the identified metabolites (Fig. 4a) (PCA model: components = 5, n = 34 (24 GFP−, 10 GFP +), number of metabolites = 58, R^2^X = 0.702, Q^2^ = 0.378). Statistical analysis was also done using OPLS-DA, and the metabolite profiles of GFP + and GFP− protoplasts could be separated even better using this method (Fig. 4b; Table 2, for loadings see Supporting Information Fig. 1) (OPLS-DA model: predictive+orthogonal components = 1 + 1, n = 35, number of metabolites = 58 and two Y–variables, R^2^X = 0.381, R^2^Y = 0.851, Q^2^ = 0.722).

**Table 2:** Metabolites found in different amounts in GFP positive (GFP+) and GFP negative (GFP ) protoplasts

The data was analysed using OPLS-DA (confidence level of parameters 99%). Shikimic acid had the highest correlation with GFP+ protoplasts, while an unknown disaccharide had the highest correlation with GFP- protoplasts. For further information about the OPLS DA model see Fig. 4b

Several metabolites were more abundant in one cell type or the other (Table 2). A group of amino acids and components of the TCA-cycle (asparagine, α-ketoglutarate, glutamine, glutarate, malate, citrate and phosphoric acid) was correlated with the GFP+ protoplasts. Asparagine has a central role in nitrogen storage and transport in plants while glutamine is associated with nitrogen assimilation and the carbon–nitrogen balance in the cell (Miflin and Habash 2002; Lea et al. 2007). Glutarate is involved in fatty acid and lysine degradation. Only an unspecified disaccharide and glycerate were positively correlated with GFP− protoplasts; the former is not known to play a prominent role in metabolism while the latter occurs in several major pathways in the plant cell.

Our data suggest that the method that we have developed is very useful for identifying metabolites that could serve as chemical markers for specific cell types within the root, and the large collections of fluorescent marked cell lines available makes it now possible to sort most types of root cells (Brady et al. 2007; Carter et al. 2013). The use of MVA to evaluate the data was essential to compare metabolite profiles in order to identify metabolites that differed significantly between root cell types. Analysing the metabolite composition of protoplasts using GC-TOF–MS will predominantly identify more highly abundant primary metabolites, but the method for protoplast isolation and sorting described here can also be combined with untargeted LC–MS analysis, thereby making it possible to detect other classes of compounds, such as polar metabolites and different secondary metabolites (Wolfender et al. 2015). Furthermore, using targeted methods of metabolite analysis, profiling of low abundant metabolites can also be performed. The availability of more general methods to identify and quantify components of primary and secondary metabolism in different cell types in a pathway context, especially in combination with information about gene expression and protein abundance, will be very important in order to increase our understanding of plant metabolism and cellular processes.

## Concluding remarks

Recent review articles have stressed the need for improved spatial resolution in metabolite analyses, going from analyses at the whole plant and tissue level to the cellular and subcellular level (Kueger et al. 2012; Sweetlove et al. 2014). Metabolite profiling of isolated cell populations and single cells has until recently been extremely difficult, but the development of ultra-sensitive mass spectrometers is now starting to make these approaches possible (Oikawa and Saito 2012).

In this study we have shown that it is possible to use protoplast isolation and cell sorting combined with sensitive mass spectrometry analyses to perform metabolite profiling of specific cell types in the *Arabidopsis* root apex. We have improved the method for root protoplast isolation and sorting, and performed a thorough validation of the method for untargeted GC-TOF–MS analysis of sorted cells. Cell type-specific targeted and untargeted MS and MS/MS analysis will be very important in increasing our understanding of cellular processes, and the method that we have developed can also be combined with protein and transcript profiling as a powerful tool for plant systems biology.

## Electronic supplementary material

Below is the link to the electronic supplementary material.

Spectral and identification information for identified metabolites, output from in-house script used for targeted analysis. Excel file containing spectral and metabolite identification information provided by the targeted analysis script (XLSX 44 kb)

Model information regarding the PCA and OPLS-DA models presented in this study (DOCX 13 kb)

Loading plots for Fig. 4. Loading plots for PCA and OPLS-DA models in Figs. 4a and 4b (TIFF 14163 kb)

Complete list of resolved metabolites (identified, classified and unknown). List of metabolites found in the data set for samples from both roots and protoplasts and their corresponding p-values from the PCA model presented in Fig. 3 (TIFF 56392 kb)
