# Supplementary material for: Cell-type specific metabolic profiling of Arabidopsis thaliana protoplasts as a tool for plant systems biology
Source: Metabolomics. 2015 Jun 6;11(6):1679–89. doi: 10.1007/s11306-015-0814-7 (PMC4605972; doi:10.1007/s11306-015-0814-7)
Supplement: Supplementary file 2 — Model information regarding the PCA and OPLS-DA models presented in this study (DOCX 13 kb) [file 11306_2015_814_MOESM2_ESM.docx]

**Supporting Information Data 2**

Model information of all PCA and OPLS-DA models presented in this work. All models were done in the SIMCA software package v. 13.0 (Umetrics, Umeå, Sweden).

**Fig. 1 a and b**

PCA model: components = 6, n = 30, number of peaks = 505, R^2^X = 0.591, Q^2^ = 0.149

**Fig. 3 b and c**

PCA model: components = 5, n = 45, number of metabolites = 175, R^2^X = 0.735, Q^2^ = 0.557

**Fig. 4 a**

PCA model: components = 5, n = 34 (24 GFP-, 10 GFP+), number of metabolites = 58, R^2^X = 0.702, Q^2^= 0.378

**Fig. 4 b**

OPLS-DA model: predictive + orthogonal components = 1+1, n= 35, number of metabolites = 58 and two Y‑variables, R^2^X = 0.381, R^2^Y = 0.851, Q^2^ = 0.722
