# Supplementary figures and images for: Cell-type specific metabolic profiling of Arabidopsis thaliana protoplasts as a tool for plant systems biology
Source: Metabolomics. 2015 Jun 6;11(6):1679–89. doi: 10.1007/s11306-015-0814-7 (PMC4605972; doi:10.1007/s11306-015-0814-7)

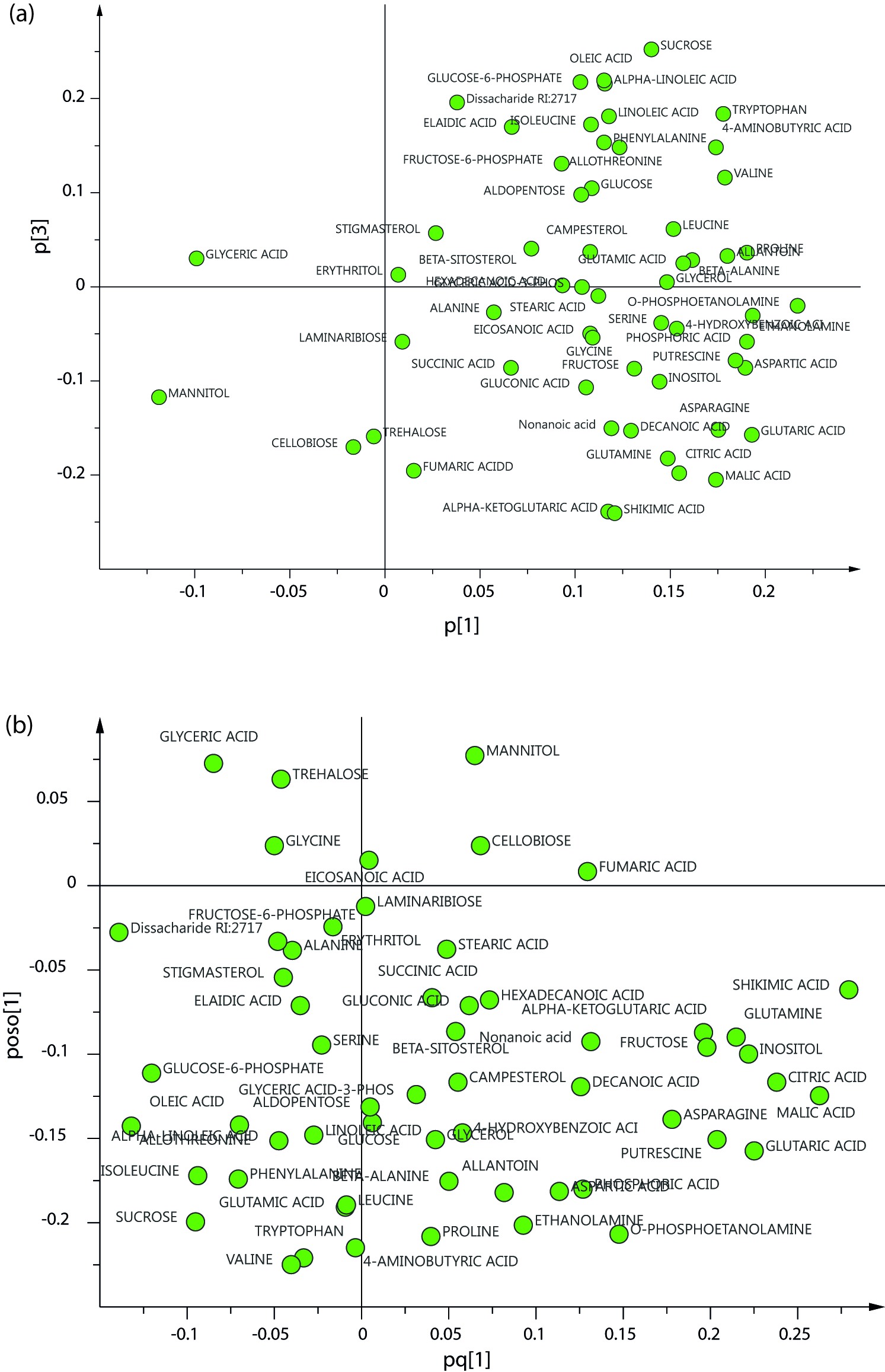

Supplement: Supplementary file 3 — Loading plots for Fig. 4. Loading plots for PCA and OPLS-DA models in Figs. 4a and 4b (TIFF 14163 kb) [file 11306_2015_814_MOESM3_ESM.tif]

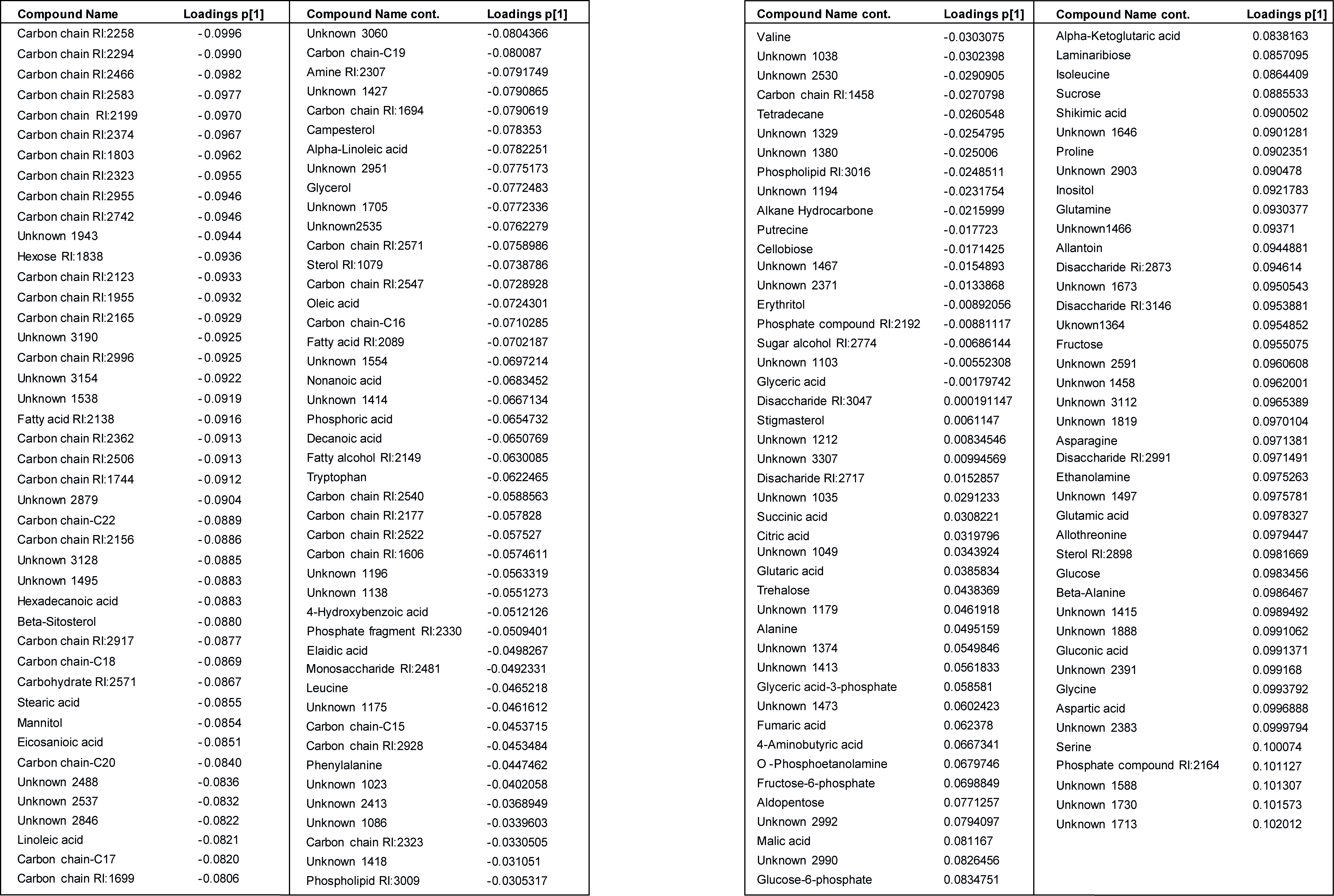

Supplement: Supplementary file 4 — Complete list of resolved metabolites (identified, classified and unknown). List of metabolites found in the data set for samples from both roots and protoplasts and their corresponding p-values from the PCA model presented in Fig. 3 (TIFF 56392 kb) [file 11306_2015_814_MOESM4_ESM.tif]
